# Social interactions predict genetic diversification: an experimental manipulation in shorebirds

**DOI:** 10.1093/beheco/ary012

**Published:** 2018-02-14

**Authors:** Charles Cunningham, Jorge E Parra, Lucy Coals, Marcela Beltrán, Sama Zefania, Tamás Székely

**Affiliations:** 1 Department of Biology, University of York, Heslington, York, UK; 2 Wildlife Conservation Society, Barrio Versalles, Cali, Columbia; 3 Milner Centre for Evolution, Department of Biology and Biochemistry, University of Bath, Claverton Down, Bath, UK; 4 Institut Supérieur de Technologie de Menabe, Port Morondava, Madagascar; 5 Department of Evolutionary Zoology and Human Biology, University of Debrecen, Egyetem tér 1, Debrecen, Hungary

**Keywords:** dispersal, genetic structure, gene flow, Madagascar, mating opportunities, mating systems, speciation, social network, spatial behavior, shorebird

## Abstract

Mating strategy and social behavior influence gene flow and hence affect levels of genetic differentiation and potentially speciation. Previous genetic analyses of closely related plovers *Charadrius* spp. found strikingly different population genetic structure in Madagascar: Kittlitz’s plovers are spatially homogenous whereas white-fronted plovers have well segregated and geographically distinct populations. Here, we test the hypotheses that Kittlitz’s plovers are spatially interconnected and have extensive social interactions that facilitate gene flow, whereas white-fronted plovers are spatially discrete and have limited social interactions. By experimentally removing mates from breeding pairs and observing the movements of mate-searching plovers in both species, we compare the spatial behavior of Kittlitz’s and white-fronted plovers within a breeding season. The behavior of experimental birds was largely consistent with expectations: Kittlitz’s plovers travelled further, sought new mates in larger areas, and interacted with more individuals than white-fronted plovers, however there was no difference in breeding dispersal. These results suggest that mating strategies, through spatial behavior and social interactions, are predictors of gene flow and thus genetic differentiation and speciation. Our study highlights the importance of using social behavior to understand gene flow. However, further work is needed to investigate the relative importance of social structure, as well as intra- and inter-season dispersal, in influencing the genetic structures of populations.

## Teaser

Polygamous Kittlitz’s plovers travel further over larger areas and interact with more individuals than the monogamous white-fronted plover. Movement patterns and social interactions influence gene flow and thus impact on speciation, although evidence from the field is limited. Here, we show that 2 Malagasy plover species exhibit different spatial behavior and social structure, consistent with existing genetic data. These results have implications for the genetic, social, and spatial processes by which new species are formed.

## INTRODUCTION

How new species emerge despite homogenising gene flow is one of the most debated topics in evolutionary biology (Price 2008; Futuyma 2013). Although speciation is possible with continuous gene flow between lineages, this typically impedes speciation (Slatkin 1987; Niemiller et al. 2008; Hereford 2009; Matute 2010; Feder et al. 2012). Understanding factors that affect gene flow is important beyond evolutionary biology; if local environments change abruptly or species suffer population or range contractions due to climate change, population fitness, and productivity may decline unless genetic diversity is preserved within the extended population (Frankham 1996; Arenas et al. 2012; Aitken and Whitlock 2013). Sexual selection, typically more intense in polygamous than monogamous species, is often considered to facilitate speciation through a variety of mechanisms via sexual conflict or intrasexual competition (Arnqvist and Rowe 2002; Ritchie 2007; Wilkinson and Birge 2010; Gavrilets 2014). Greater gene flow creates more uniform population genetic structure, but it also maintains greater genetic diversity within the population (Aitken and Whitlock 2013; Eberhart-Phillips et al. 2015). However, recent work suggests that the variance in mating success associated with strong sexual selection may also constrain speciation through promoting individual spatial movement, resulting in increased gene flow in polygamous species (Küpper et al. 2012; D’Urban Jackson et al. 2017).

Dispersal events typically increase gene flow, including natal and breeding dispersal, migration, as well as fine-scale movements that increase demographic connectivity within populations (Ronce 2007; Pilot et al. 2010; McGuire 2013; Burns and Broders 2014). Many species of birds and mammals disperse to enhance mating opportunities and reproductive success; and access to mates, resources, and the avoidance of inbreeding are important in promoting sex-specific dispersal (Greenwood 1980; Lenormand 2002; Trochet et al. 2016). However, fine-scale continuous events, such as the social environment, spatial distribution, and mate search behavior, are often overlooked (Skrade and Dinsmore 2010; Wey et al. 2015) in favor of rarer, large-scale dispersal events which cannot explain observed levels of gene flow alone (D’Urban-Jackson et al. 2017, Morinha et al. 2017). Individual movement patterns and space use strategies can influence social interaction as well as mating success, and hence gene flow (Duvall 1997; Sih et al. 2009; McGuire 2013). As well as affecting gene flow, the spatial distribution of individuals may in turn influence encounter rates impacting sexual competition (Tuni and Berger-Tal 2012; D’Urban Jackson et al. 2017). This alteration of sexual selection patterns will in turn influence mating strategies (Oh and Badyaev 2010), which provides feedback into movement patterns (Fromhage et al. 2016). Additionally, studies of social behavior in birds, insects, and mammals have predicted higher levels of social interaction result in more gene flow, less speciation, and higher extinction rates (Cockburn 2003; Wilkinson and Birge 2010; McGuire 2013); suggesting gene flow may be reduced through limited social interactions.

Recent genetic analyses of closely related shorebirds, the Kittlitz’s plover *Charadrius pecuarius* and the white-fronted plover *Charadrius marginatus*, showed that they exhibit different population genetic structure throughout their breeding range in Madagascar: Kittlitz’s plover had a panmictic and homogenous population with no population structure detected, whereas the white-fronted plovers exhibited well-defined geographically distinct populations (Eberhart-Phillips et al. 2015). The life-history and ecology of these 2 species are very similar, e.g., both are small insectivorous ground-nesting shorebirds with modal clutch size of 2 eggs and precocial young, and these species often breed side by side in Madagascar (Zefania and Székely 2013). However, their mating systems are different: Kittlitz’s plovers are polygamous whereas white-fronted plovers are socially (and genetically) monogamous (Zefania et al. 2010; Maher et al. 2017). Parra et al. (2014) found that remating times were different between male and female Kittlitz’s plovers, whereas in white-fronted plovers the remating times were similar for males and females, demonstrating interspecific variation in mating opportunities and mate fidelity. The genetic data on population structure across a large geographic area (Eberhart-Phillips et al. 2015) and the experimental manipulation of mating opportunities in the field (Parra et al. 2014) provide a unique opportunity to explore the spatial and social processes through which sexual selection may influence gene flow within breeding seasons by using data that have not been analyzed previously.

Here, we investigate movement and interaction of experimental plovers, using spatial and network methodologies to analyze experimental data, to test 2 key predictions. First, due to differences in mating opportunities, we predicted more movement by polygamous Kittlitz’s plovers in order to find new mates compared with monogamous white-fronted plovers (Székely and Lessells 1993; Küpper et al. 2012; Parra et al. 2014), specifically greater distance travelled over larger home ranges as well as higher dispersal distance. Second, in accordance with the first prediction and known population structure (Eberhart-Phillips et al. 2015), Kittlitz’s plovers should demonstrate greater spatial and social interaction with conspecifics than white-fronted plovers. Plovers have often been used as a behavioral model system to understand mating system evolution (Székely et al. 2006; Vincze et al. 2016; Maher et al. 2017), and testing these predictions using spatial and social interaction data will provide the link between population genetic study and diversification, and mating system variation using the Malagasy plovers as a case study.

## METHODS

### Study species and study sites

**Figure 1:**
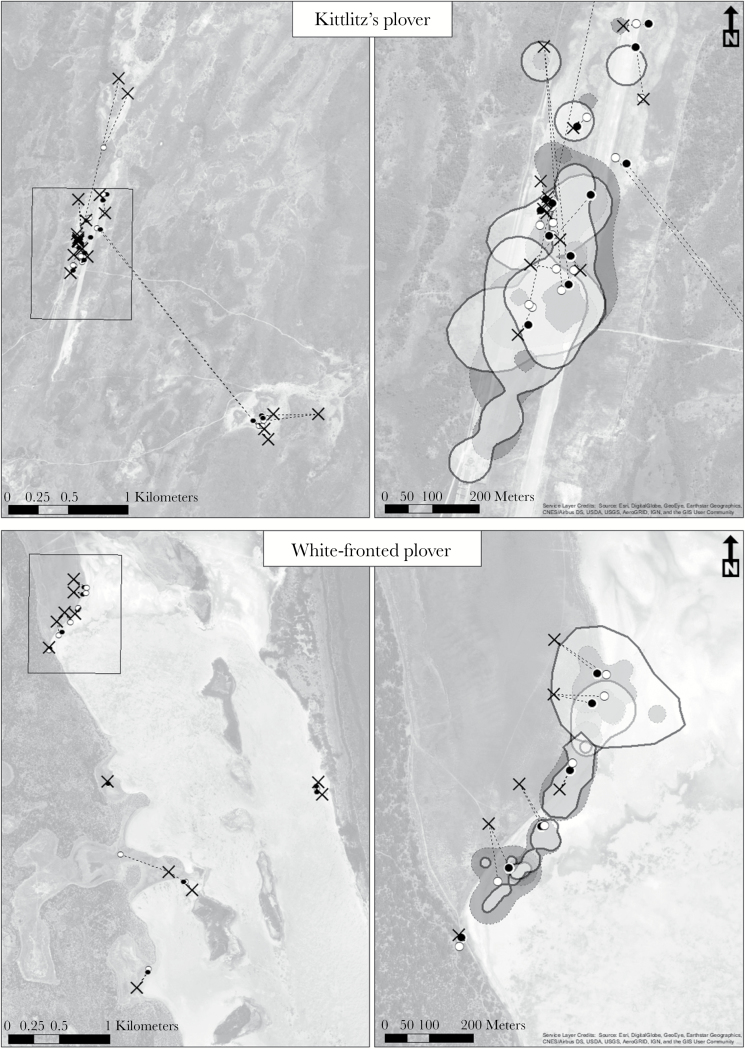
Study sites of Kittlitz’s and white-fronted plover in SW Madagascar, with the study area in the left panel and illustrative detail in the right. Dashed lines represent the breeding dispersal between the original nest capture sites (denoted by crosses), and secondary territories of male (white circles) and female (black circles) experimental plovers used in the spatial analyses. As an illustration of data used in analysis, the home ranges of 3 male (white fill, solid outline) and female (grey fill, dashed outline) Kittlitz’s plovers and 5 white-fronted male and female plovers are shown.

Kittlitz’s and white-fronted plovers were investigated in southwest Madagascar. Kittlitz’s plovers were studied between 6 February and 13 May 2010 in Andavadoaka (22° 02′S, 43° 39′E, Figure 1) where approximately 300 Kittlitz’s plovers breed around alkaline lakes (J.E. Parra, S. Zefania, and T. Székely, unpublished data). Fieldwork with the white-fronted plover was carried out between 1 April and 23 June 2011 at Lake Tsimanampetsotsa National Park (24° 3′S, 43°44′E, Figure 1), a large alkaline lake (15 km × 0.5 km), surrounded by sandy beaches, short grass, and saltpans. Approximately 150 white-fronted plovers breed around the lake (J.E. Parra, unpublished data).

In the field, nests were searched for on foot or from hides by spotting incubating parents returning to their nest. In total, 18 Kittlitz’s plover pairs (36 individuals) and 14 white-fronted plover pairs (28 individuals) were captured with funnel traps placed on their nests (Figure 1). The differing sample sizes reflect the maximum number that was possible to catch with the resources available (J.E. Parra, S. Zefania, & T. Székely, unpublished data). Nest search, trapping, and behavioral observations followed standard protocols that have been adopted in previous publications (Székely et al. 2008; Carmona-Isunza et al. 2015; Vincze et al. 2016; Maher et al. 2017). The traps were continuously monitored until a parent entered the trap and sat on the eggs, and then it was removed immediately to reduce stress and the risk of injury. All adults were ringed with an individual color ring combination and a numbered SAFRING metal ring from the University of Cape Town, South Africa. Study birds were differentiated from other ringed individuals by using green permanent marker (Pilot Supercolour) on the individual’s white belly.

### Mate-removal experiment

The mate removal protocol of Székely et al. (1999) was followed to experimentally create unmated sexually-active individuals. This experimental treatment ensured that a mate-searching phase was included within the movement of all individuals, which would not have been possible with purely observational study. Briefly, both parents were trapped, ringed, measured and a blood sample was taken for sex determination since the adult plumage is sexually monomorphic in both species (see Supplementary Information). One parent was then selected at random (since the sex was not known until after the experiment) for release at the capture location immediately; and the other parent was taken into captivity. In both Kittlitz’s and white-fronted plovers, both the male and female incubate the eggs (Urban et al. 1986; Hockey et al. 2005). Eggs of experimental birds were translocated to nearby conspecific nests with eggs at a similar developmental stage. Only pairs incubating 2 eggs (modal clutch size in both species) were manipulated. Trapping locations for both species were distributed evenly over an area of similar size (Figure 1).

Removed plovers were transported to a nearby purpose-built aviary as detailed in Parra et al. (2014). Captive plovers were measured and then released after their former mate either found a new mate, or was not seen in the study sites for at least 12 days. Time in captivity was comparable for white-fronted plovers (number of days in captivity: 8.0 ± 1.71 days, *N* = 14, we provide mean ± SD unless stated otherwise) and Kittlitz’s plovers (7.12 ± 2.57 days, *N* = 18). Although captive plovers appeared to lose a small amount of body mass during their time in captivity (2.77 ± 0.51 g in Kittlitz’s plover, and 0.73 ± 0.22 g in white-fronted plover), many remated shortly after release indicating salubrious condition. The experiment was approved by the Malagasy authorities(see Ethical Note) .

### Behavioral observations

Both the immediately released and the captive plovers released from the aviary were searched for within the study area every day in the field, using a car and mobile hide, after release. When an experimental plover was found, the coordinates of its location were taken with a handheld GPS receiver (Garmin e-Trex H). In addition, we collected 30-min behavioral samples of one of the 2 species, the white-fronted plover, by recording the behavior of experimental plovers every 30 s (see details in Parra et al. 2014) immediately after a resighting. The identity of other experimental plovers the focal individual interacted with during this time was also recorded. Although attempted, it was not possible to complete 30-min behavioral samples for every resighting due to logistical survey limitations, i.e., if the focal individual flew off. Behavioral categories included social interactions such as fighting, courting and copulation. Two observers (M.B. and J.E.P.) collected the behavioral records, and both sampling methodology and behavioral categories were standardized between the observers. Since adults are sexually monomorphic in both species (Urban et al. 1986; Hockey et al. 2005) we used molecular sex typing to determine the sex of individuals (dos Remedios et al. 2010). Molecular sexing was carried out in NERC-Biomolecular Analysis Facility at the University of Sheffield (for details see dos Remedios et al. 2010; Parra et al. 2014, Supplementary Information).

### Home range and movement analyses

The R package *adehabitatHR* (Calenge 2006) was used to calculate the home ranges of individual plovers using the kernel method (Worton 1989) using every observed sighting of each individually marked plover (termed “relocations” henceforward). First, the utilization distributions (UD) of 24 white-fronted (total relocations 327; mean 13.63 ± 5.75, min 6, max 26) and 32 Kittlitz′s plovers (total relocations 512; mean 16.0 ± 6.53, min 6, max 28) were calculated; 2 white-fronted and 4 Kittlitz’s individuals were not included in the UD analyses because they had less than 6 relocations (Calenge 2006). The kernel smoothing parameter, h, was optimized by the least-square cross validation (LSCV) method (Gitzen and Millspaugh 2003). For several individuals, the LSCV did not converge (Seaman and Powell 1998), hence in order to produce a UD for every experimental individual, smoothing parameter limits were set beyond which the ad hoc method was used (Worton 1995; Calenge 2011; Kie 2013). These limits were set by eye to ensure there was not unrealistic fragmentation or over-smoothing of home ranges. The home range was then calculated from the UD as the area within which the probability of locating an individual is equal to a specified value (Worton 1989, Calenge 2011). To include the mate searching area as well as the core use area, a 90% home range was used in the analysis (Figure 1, Supplementary Figure S1) as it provided the largest reliable home range size (Börger 2006).

Second, plover movement was investigated using step lengths of individuals (Marsh and Jones 1988; Turchin 1998; Zeller et al. 2012), calculated with the R package *adehabitatLT* (Calenge 2006). Step lengths, calculated as the distances between consecutive points (Figure 1, Supplementary Figure S1), were summed and then divided by the number of relocations to infer the mean step length for each individual Kittlitz’s plover (grand mean step duration: 1.88 ± 0.77 days) and white-fronted plover (1.04 ± 0.68 days). Third, breeding dispersal was investigated as the net distance between territories (Figure 1, Supplementary Figure S1. This was calculated from the distance between the first nest location in the original territory, i.e., the capture point; and the centroid point of the core-use area, i.e., the secondary territory. The core-use area was calculated as the 50% home range using the same technique used to find the 90% home range size .

Generalized linear models (GLMs), with Gaussian error structure and identity link function, were used to test whether species, sex, and their interaction predict the spatial behavior of plovers (i.e., home range size, mean step length, and distance from previous territory). Log transformation was used for each response variable to normalize the data. Two models were fitted for each predictor variable, one basic model with fixed factors of species, sex, and species × sex interaction; and another model with additional control variables including number of days tracked, number of relocations, and captivity (i.e., released immediately after capture in the field, or released from captivity). “Number of relocations” was not included in the GLM analyses of mean step length as it was used in the calculation of the variable. The models were compared using an Analysis of Deviance test, and in all cases the more complex model did not improve the fit of the basic model (see Supplementary Information), and so the basic models were retained.

### Spatial interaction analyses

Spatial interaction between experimental plovers was estimated, using the Utilization Distribution Overlap Index (UDOI) with the R package *adehabitatHR* (Calenge 2011), as a proxy for behavioral connectivity within plover populations: greater overlap between home ranges indicates higher levels of space sharing and greater opportunity for social interaction, and potentially, increased gene flow through the population. The UDOI is an estimate for space use sharing between individuals (Fieberg and Kochanny 2005; Chynoweth et al. 2015), which utilizes the UD (see Home range and movement analyses). Thus, UDOI indices were calculated between the UD of individual plovers monitored during the study period. UDOI values range from 0.0 to 2.0, a value less than one indicates less overlap than expected whereas a value above one indicates higher overlap than would be expected relative to uniform space use (Fieberg and Kochanny 2005). All interactions were temporally constrained, so that interactions between experimental individuals that had no temporal overlap in relocations were not included in the analysis. Although the 2 species had different numbers of individuals for the interaction analyses (32 Kittlitz’s and 24 white-fronted plovers) and so UDOI could not be directly compared, we calculated 2 further characteristics using UDOI: 1) interaction network density, and 2) relative spatial overlap between sexes. These measures are suitable for comparison as they describe overall network structure and are not affected by group size (Wey et al. 2008).

The spatial interaction network 1) was produced using the R package *igraph* (Csardi and Nepusz 2006). For each species, an interaction matrix was created of UDOI weighted ties (edges) between individuals (nodes) where UDOI was positive. Thus, a node represents an individual plover, and edges represent its spatial interactions between individuals within the sampled population. Network density was then calculated as the proportion of potential edges, i.e., all of the possible interactions, which were observed in the network, i.e., UDOI greater than 0 (Wey et al. 2008). Standard errors for the species interaction network densities were calculated using 9999 network bootstraps (Snijders and Borgatti 1999), and 2 sample *t*-tests were carried out to test whether interaction networks significantly differed between species.

To test whether the spatial interaction network functioned as a suitable proxy for behavioral connectivity, a social interaction matrix was created using behavioral observations of experimental white-fronted plovers after release. Edges were weighted by the number of 30-s intervals in which either courtship or fighting behavior was recorded with other experimental individuals, during the 30-min observation period taken after each relocation. The observed interaction matrix was then compared to the UDOI matrix using a partial Mantel test, utilizing the R package *ecodist* (Goslee and Urban 2007). The distances between territories, i.e., the centroid point of the 50% home range core-use area (see Home range and movement analyses), of individuals were included as the control matrix. Data were not available to create a behavioral interaction matrix for Kittlitz’s plover.

As overlap size is dependent on the number of conspecific experimental individuals within the study area, it is not possible to compare overlap, i.e., the UDOI value, directly between groups of differing network sizes, unlike network density. Hence sex-specific interactions 2) were investigated separately for each species; total spatial interaction between individual plovers and either conspecific males, or females, was calculated. GLMs with Gaussian error structure and identity link function were fitted with sex as a predictor variable, and response variables of total UDOI between the focal bird and all individually marked males, and then all individually marked females involved in the study. The response variables, Y, were transformed to a normal distribution by adding one and then log transforming, i.e., ln(Y_i_ + 1).

Spatial analysis was carried out in R (R Core Team 2015) and ArcGIS 10.4 (Esri, Redlands, CA), and spatial data was converted between them using R packages *maptools* (Bivand and Lewin-Koh 2017) and *rgdal* (Bivand et al., 2017). Figures were produced using the R packages *ggplot2* (Wickham, 2009) and *igraph* (Csardi and Nepusz 2006). 

### Ethical note

Both experiments were approved by the Ministry of Environment, Forests and Tourism of the Republic of Madagascar (Research permit No: 053/11/MEF/SG/DGF/DCB.SAP/SCB of 11 March 2011 and 132/10/MEF/SG/DGF/DCB.SAP/SSE of 6 May 2010) and Madagascar National Parks (No: 398-10/MEF/SG/DGF/DVRN/SGFF of 18 May 2011). Blood sampling was also covered by these research permits. The blood transport permit was approved by Service de la Gestion de la Faune et de la Flore, Direction de la Valorisation des Resources Naturelles, Ministère de l’Environnement et des Forêts Madagascar (authorization number 080N-EA06/MG11). Kittlitz’s and white-fronted plovers are common breeding birds in much of Africa and Madagascar and not considered threatened (IUCN 2017).

The experiment was designed to reduce adverse effects on local plover populations and all necessary precautions were taken to ensure their welfare was suitably protected. Captive plovers were monitored daily and kept under standard conditions (see Parra et al. 2014) to reduce their stress levels. In addition, translocated eggs coped with the natural breeding conditions of local clutches in the 2 plover populations. Although monitoring the augmented clutches was beyond the scope of the experiment, nest checks suggest that at least 33.3% and 19.4% of augmented nests survived until hatching in the Kittlitz’s plover (*N* = 36 nests) and the white-fronted plover (*N* = 20 nests), respectively. Survival in these nests appeared to be higher than for unmanipulated nests (13.4% and 8.9%, based on *N* = 101 Kittlitz’s plover nests and *N* = 56 white-fronted plover nests, respectively; J.E. Parra et al., unpublished data).

## RESULTS

### Home range size and movement

**Figure 2:**
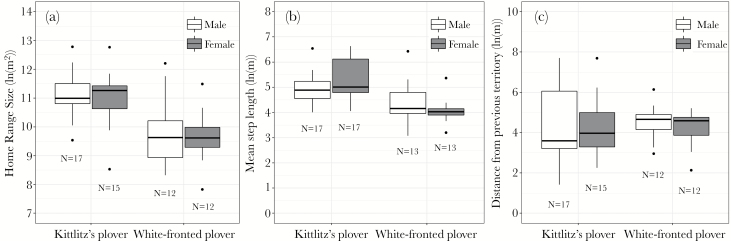
(a) Home range size, (b) mean step length and (c) dispersal from previous territory in 2 Malagasy plover species. The data were normalized using natural log transformations. Numbers of individuals are shown beneath categories. The lower and upper borders of the box are lower and upper quartiles, respectively; the horizontal bar is the median; and whiskers represent the lowest and highest observations apart from the outliers. Circles denote outliers that are above or below the interquartile range multiplied by 1.5.

Kittlitz’s plovers had significantly larger home ranges (9.02 ± 8.21 ha, *N* = 32 plovers) than white-fronted plovers (3.27 ± 4.74 ha, N = 24 plovers; Table 1), although home range sizes did not differ between males and females (Figure 2, Table 1). Kittlitz’s plovers also had a higher mean step length (223.8 ± 194.1 m, *N* = 34 plovers) than white-fronted plovers (94.0 ± 117.3 m, *N* = 26 plovers), and a marginally significant species * sex interaction suggests sex-difference between the 2 species (Table 1). Although the mean step duration was longer in Kittlitz’s plover (2 sample *t*-test: *t*_54_ = 3.84, *P* < 0.001), days of tracking (duration) did not explain significant variation in mean step length (Supplementary Table S2).

**Table 1:** General linear models of home range size, mean step length and dispersal from previous territory (response variables) of male and female Malagasy plovers. Figures in bold indicate statistically significant relationships. The data were natural log transformed prior to the analyses

	Home range size		Mean step length		Dispersal from previous territory	
	Estimate	*t* value	Estimate	*t* value	Estimate	*t* value
Intercept	11.104 (0.241)	46.092 (<0.001)	4.978 (0.171)	29.202 (<0.001)	4.354 (0.336)	12.960 (<0.001)
Species	-1.264 (0.375)	**-3.374 (0.001**)	-0.598 (0.259)	**-2.310 (0.025**)	0.160 (0.522)	0.305 (0.761)
Sex	-0.074 (0.352)	-0.211 (0.833)	0.315 (0.241)	1.307 (0.197)	-0.051 (0.491)	-0.103 (0.918)
Species × Sex	-0.121 (0.537)	-0.225 (0.823)	-0.647 (0.366)	-1.767 (0.083)	-0.242 (0.749)	-0.323 (0.748)

General linear models using Gaussian error structure and identity link function were fitted separately to home range size, mean step length and dispersal from previous territory. These models were compared with models including additional control variables; these were found not to improve the model fit, and hence the more basic models were sufficient (see Supplementary Information). Standard errors and p-values are in presented in parentheses for the estimates and t-values, respectively. “Female,” “white-fronted,” and “white-fronted female” were used as reference levels.

Contrary to expectations, the distance from the former territories to the new territories was not different between Kittlitz’s plovers and white fronted plovers, nor did it differ between males and females (Table 1). However, Kittlitz’s plovers were found to have greater variation in their breeding dispersal distances than white-fronted plovers (*F*-test: *F*_31,23_ = 0.290, *P* = 0.003, Figure 2).

### Spatial interaction

**Figure 3:**
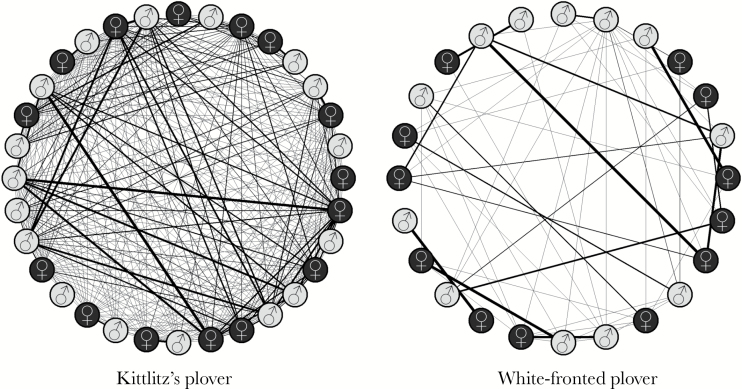
Spatial association networks of Kittlitz’s and white-fronted experimental plovers. Nodes represent adult males and females; vertices represent the amount of overlap (UDOI) of individual’s home ranges. The Kittlitz’s network was more interconnected than the white-fronted network, as the densities differed significantly (2 sample *t*-test, *t*_54_ = 4.462, *P* < 0.001).

Kittlitz’s plovers were more spatially interconnected than white-fronted plovers. The density of the Kittlitz’s plover spatial association network (0.742 ± 0.093 [SE], *N* = 32 plovers) was significantly higher (2 sample *t*-test: *t*_54_ = 4.399, *P* < 0.001, Figure 3) than that of the white-fronted (0.284 ± 0.047 [SE], *N* = 24 plovers). Additionally, the white-fronted spatial association network was significantly correlated with the observed behavioral interaction network (partial Mantel test: *r*_m_ = 0.351, *P* < 0.001) controlling for distance between territories.

**Figure 4:**
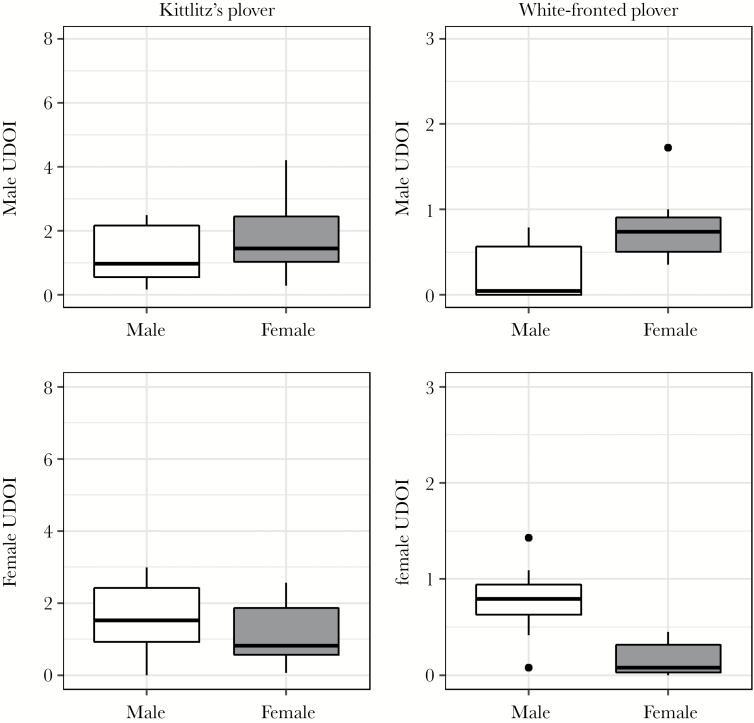
Total spatial overlap of individual home ranges with conspecific experimental males or females in white-fronted and Kittlitz’s plover, quantified using the utilization distribution overlap index (UDOI). Interspecies comparison of UDOI size is not appropriate due to differing sample sizes, but the relationship between male and female overlap within species can be compared. The lower and upper borders of the box are lower and upper quartiles, respectively; the horizontal bar is the median; and whiskers represent the lowest and highest observations apart from the outliers. Circles denote outliers that are above or below the interquartile range multiplied by 1.5.

The spatial interactions of Kittlitz’s plovers were less sexually structured than those of white-fronted plovers (Figure 3). In Kittlitz’s plover, an individual’s spatial overlap with both males and females was not predicted by the sex of the interacting individual indicating a lack of sex-specific spatial interactions (GLMs, males: *t* = 1.633, *P* = 0.113; females: *t* = 1.341, *P* = 0.190; Figure 4; Table 2). In contrast, male white-fronted plovers had more spatial interaction with females than males (GLM: *t* = 4.137, *P* < 0.001; Figure 4). Likewise, female white-fronted plovers had a larger amount of spatial interaction with males than females (GLM: *t* = 5.652, *P* < 0.001; Figure 4; Table 2).

**Table 2:** General linear models of total spatial overlap of the focal individual with males, and females (response variables) in 2 species of Malagasy plovers. Figures in bold indicate statistically significant relationships. The data were natural log transformed prior to the analyses

		Male overlap		Female overlap	
		Estimate	*t* value	Estimate	*t* value
White-fronted	Intercept	0.191 (0.062)	3.061 (0.006)	0.554 (0.052)	10.642 (<0.001)
	Sex	0.365 (0.088)	**4.137 (<0.001**)	-0.416 (0.074)	**-5.652 (<0.001**)
Kittlitz’s	Intercept	0.733 (0.097)	7.531 (<0.001)	0.896 (0.095)	9.418 (<0.001)
	Sex	0.232 (0.142)	1.633 (0.113)	-0.186 (0.139)	-1.341 (0.190)

General linear models using Gaussian error structure and identity link function were used to analyse spatial overlap. As interspecies analysis was not appropriate due to differing sample sizes, the only explanatory variable included was Sex, with *Female* the reference factor level. Estimate standard errors and *t* values’ corresponding *P*-values are in parentheses.

## DISCUSSION

The analyses of experimental data that have not been presented previously showed that spatial movement and interaction of unmated individuals varies between closely-related species. These results augment the analyses of Parra et al. (2014) that reported different mating times, courtship behavior and pair bonds between the 2 Malagasy plover species. The work presented here provided 2 novel results; species differences in both spatial behavior and inferred social interactions with conspecifics.

Firstly, both mean step length and home range size were larger in Kittlitz’s than in white-fronted plovers. This result showed that the polygamous Kittlitz’s plovers exhibit less restricted movements than the socially (and genetically) monogamous white-fronted plover. The smaller home ranges in white-fronted plover may be due to more restricted mate search behavior and/or to more limited movements of experimental birds once they found a mate. We believe both explanations are likely and more work is needed to disentangle the movements of unmated and mated individuals possibly by recording the movements of radio-tagged individuals. Furthermore, although field observations were carried out as consistently as possible between the 2 species, the relocations were not necessarily uniformly spaced between the 2 species.

Contrary to expectation, new Kittlitz’s and white-fronted plover territories were similar distances from their previous territories, and hence the breeding dispersal distances do not seem to explain differences in gene flow within these populations of plovers. However, the other measures of spatial distribution and search effort did align with the observed genetic structure, and for future studies we recommend using territorial or social metrics alongside distance metrics where possible to understand fine-scale spatial patterns. Other studies have also found social interactions and spatial behavior explaining gene flow, in addition to or in absence of dispersal (Burland et al. 1999; Pilot et al. 2010; McGuire et al. 2013). Although migration did not predict genetic differentiation in shorebirds (D’Urban-Jackson et al. 2017) it remains important to consider the role interseason movement plays, such as natal dispersal (Ronce 2007; Mabry et al. 2013), which is not investigated in this study. Further work is needed to distinguish the relative importance of natal versus breeding dispersal in generating gene flow (Wey et al. 2015). Importantly, although there was no difference in the dispersal distance between the species, Kittlitz’s plovers showed greater variation in their dispersal distances: the furthest Kittlitz’s plover dispersed was 2202m, compared to 462m in white-fronted plover. This demonstrates the capability to disperse greater distances within a breeding season, and deserves further study in the context of other forms of dispersal.

Second, consistent with our predictions, Kittlitz’s plovers were more spatially interconnected than white-fronted plovers. The Kittlitz’s plover spatial interaction network density was significantly higher, and therefore birds likely interact with higher numbers of conspecific experimental individuals. The spatial association network of white-fronted plover correlated with the observed social interaction network, indicating that results from the spatial interaction network may also be considered in the context of a social network.

In Kittlitz’s plover, home ranges of both males and females overlapped with several other experimental individuals. The high levels of spatial interaction suggest flocking behavior; Kittlitz’s plovers exhibit complex gregarious social behavior where individual plovers join to flock for feeding and resting; even members of breeding pairs join flocks (Urban et al. 1986; Hockey et al. 2005), but the relative numbers of paired and unpaired individuals within these flocks is not currently known. This greater degree of sociality increases the potential for high levels of gene flow across a population; however, a recent study of genetic structure in the social, but monogamous, red-billed chough *Pyrrhocorax pyrrhocorax* found strongly segregated populations (Morinha et al. 2017), suggesting both mating opportunities *and* social interaction are needed to facilitate high levels of gene flow. Flocking behavior may facilitate gene flow through lower energy costs associated with mate searching due to high densities, and reduced risk of predation while searching (DeRivera et al. 2003; Kasumovic et al. 2007).

Kittlitz’s plover’s exhibit uni-parental brood care, whereas white-fronted plovers are biparental (Zefania and Székely 2013), and this brood care strategy may allow them to interact more frequently with other members of the population due to less time spent on parental care. In contrast, white-fronted plovers exhibit greater philopatry and are less social when searching for a mate, and males and females had few interactions with conspecifics, inherently leading to strongly spatially structured populations. This suggests polygamous plover species have a plastic, flexible social structure which spreads over a broad geographical range (Küpper et al. 2012; Eberhart-Phillips et al. 2015), whereas monogamous plovers exhibit social rigidity with few social interactions within a restricted home range. A recent genetic analysis of 79 geographically distinct populations of 10 plover species provided consistent results with the latter argument, since polygamous plovers exhibited less geographic differentiation than monogamous ones (D’Urban Jackson et al. 2017).

Additionally, interactions between plovers were not sexually structured in Kittlitz’s plovers, but were in white-fronted. We found that a significant difference in spatial overlap between the sexes was only found in white-fronted plovers; overlap with the opposite sex was significantly higher than same sex overlap for both males and females. Small home range overlap with same-sex individuals, combined with the previous results of fewer interactions and less movement, suggests strong territoriality in white-fronted plovers (Ostfeld 1986). This fits in with the expectation that pair bonding and biparental care will generally see an individual be more fixed in its range of movement (Fricke 1986; Sommer 2003). In line with the latter argument, female pied flycatchers *Ficedula hypoleuca* exhibit similar restricted mate searches as a consequence of competition with conspecifics for nest sites (Slagsvold et al. 1988).

An explanation of the spatial patterns observed in white-fronted plover may be the costs of finding nest sites leading to high territoriality (Brashares and Arcese 1999). Strong competition for breeding vacancies would prevent formation of a floating population of single plovers as any paired plover has a high probability of losing their breeding status if they leave a nesting territory in an attempt to find a new mate. Therefore, if the costs of searching are related to defending a nesting site, males and females should stay together to protect a territory and spend less time searching for a mate to reduce the risk of losing both their nest site and breeding status (Ens et al. 1996). White-fronted plovers consistently exhibit high breeding site-fidelity and territory retention within and between years (Lloyd 2008). Consequently, monogamy and biparental care associated with territoriality are probably the best strategy to maximize reproductive success in white-fronted plovers as demonstrated in other shorebird species (Lessells 1984; Gratto et al. 1985). Conversely, breeding sites do not seem to be limited for Kittlitz’s plovers, they can breed with nests of different pairs 10–30 m apart (Urban et al. 1986; Hockey et al. 2005). Hence, the observed tight sexually-structured spatial behavior in white-fronted plovers may be indicative of territoriality, resulting in low gene flow through a population due to confined search behavior and low interaction rate.

In conclusion, we found different spatial movements and inferred social interaction patterns in unmated individuals of closely related plover species exhibiting different breeding systems. These findings may have important implications for the role of spatial interaction in gene flow and speciation; as well as how spatial behavior and social interactions are shaped by competition, mating opportunities and territoriality. Taken together, the different spatial behavior and social structure in 2 sympatric plover populations are consistent with molecular results obtained from populations along the west coast of Madagascar (Eberhart-Phillips et al. 2015). Our study demonstrates that spatial and network analyses provide valuable tools in investigating, and quantifying, how social interaction, competition and mating strategies impact on gene flow and speciation. In particular, we emphasize the need for social and/or territory metrics to be used in accordance with distance metrics when investigating genetic structure. Future investigations with detailed movements of focal species, in an explicit phylogenetic framework, are needed to fully understand the roles of mating system and social interaction, as well as the relative importance of intra- and interseason movements in speciation.

## SUPPLEMENTARY MATERIAL

Supplementary data are available at *Behavioral Ecology* online.

Supplementary Figure S1

Supplementary Information
