# Supplementary figures and images for: Social interactions predict genetic diversification: an experimental manipulation in shorebirds
Source: Behav Ecol. 2018 Feb 14;29(3):609–18. doi: 10.1093/beheco/ary012 (PMC5946871; doi:10.1093/beheco/ary012)

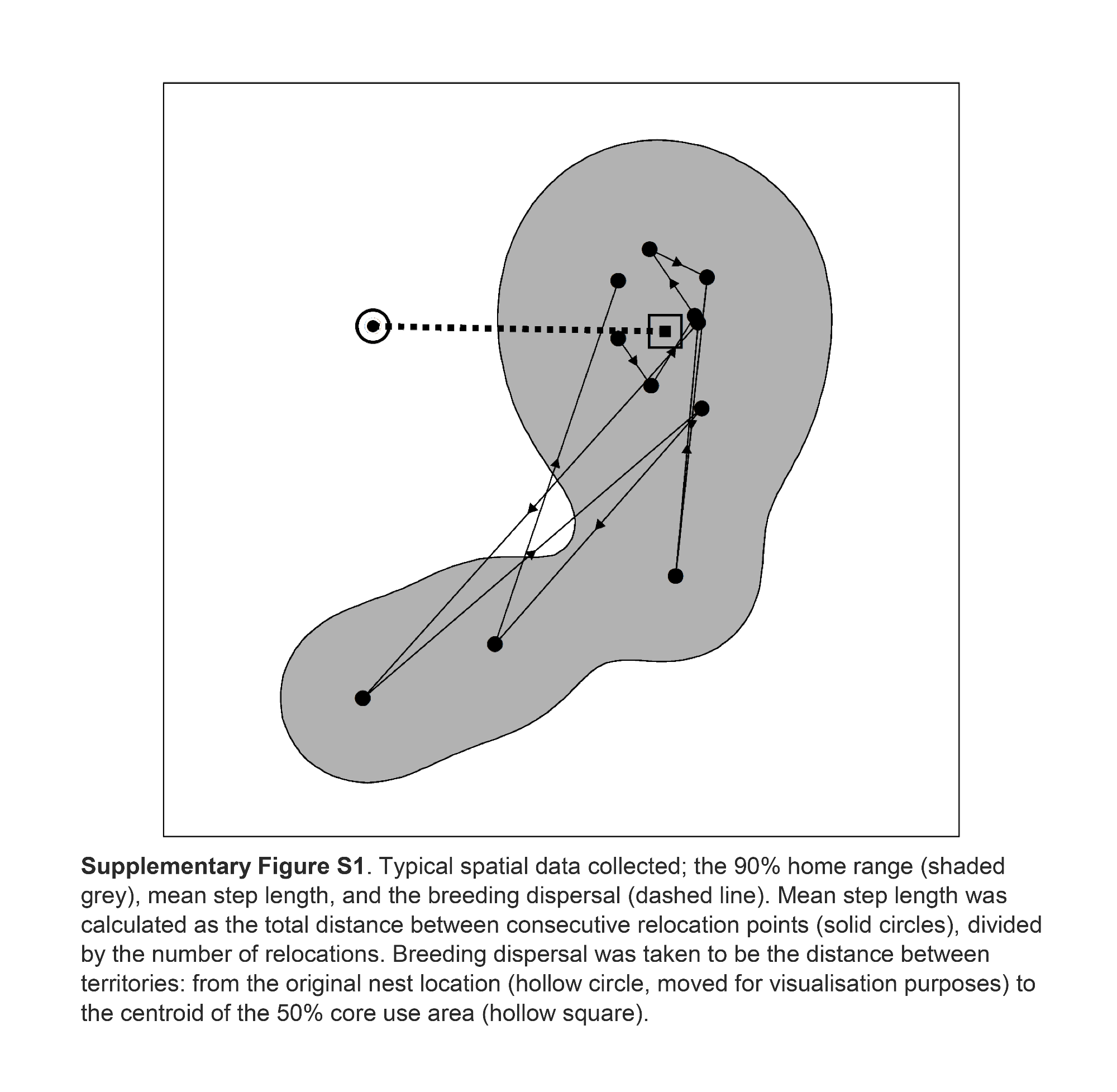

Supplement: Supplementary Figure S1 [file ary012_suppl_supplementary_figure_s1.png]
