# Supplementary material for: Social interactions predict genetic diversification: an experimental manipulation in shorebirds
Source: Behav Ecol. 2018 Feb 14;29(3):609–18. doi: 10.1093/beheco/ary012 (PMC5946871; doi:10.1093/beheco/ary012)
Supplement: Supplementary Information [file ary012_suppl_supplementary_information.docx]

Supplementary Information

*Molecular sexing*

A small blood sample was taken from each adult's brachial wing vein, by puncturing, collecting drops of blood (25 μl approx.) in capillary tubes, and storing this in Eppendorf tubes of Queen's Lysis Buffer. DNA was extracted from blood samples using the ammonium acetate extraction method (Parra et al., 2014). For molecular sex typing, Z- and W-chromosome-specific genes were amplified via polymerase chain reaction (PCR) using Z-002B/Z-002D primers. For additional certainty in sex assignment, W-chromosome-specific Calex-31 primers, developed in the genus *Charadrius*, were utilized (Parra et al., 2014). PCR amplification was conducted on a DNA Engine Tetrad 2 Peltier Thermal Cycler under the following conditions: 95 °C for 15 min, followed by 35 cycles of 94 °C for 30 s, 56 °C for 90 s, 72 °C for 60 s with a final extension of 60 °C for 30 min. Samples were visualized on an ABI 3730 automated sequencer. IR Dye-labelled tailed primers separated the products of Z-002B/Z-002D primers into either one (ZZ) or two (ZW) bands, indicating male or female, respectively. The W-specific Calex-31 product only appeared as one band indicating female. Images were scored using GeneMapper software version 4.1 (Applied Biosystems, Foster City, CA, U.S.A.). To maximize reliability, all samples were sexed using two markers. Additionally, for 8% of the total samples (11 Kittlitz's plover and 10 white-fronted plover individuals) molecular sexing was repeated; in all cases, repetitions concurred with the original results.

**Table S1.** Home range sizes of male and female Malagasy plovers.

|  | Model 1 | | Model 2 | |
| --- | --- | --- | --- | --- |
|  | Estimate | t value | Estimate | t value |
| Intercept | 11.104  (0.241) | 46.092  (< 0.001 ***) | 11.526 (0.539) | 21.396  (< 0.001 ***) |
| Species  (white-fronted) | -1.264  (0.375) | -3.374  (0.001 **) | -1.725  (0.427) | -4.038  (< 0.001 ***) |
| Sex  (female) | -0.07438  (0.352) | -0.211  (0.833) | 0.054 (0.34352) | 0.158  (0.875) |
| Number of relocations | - | - | 0.027  (0.025) | 1.075  (0.288) |
| Captivity  (yes) | - | - | 0.195 (0.274) | 0.714  (0.479) |
| Duration | - | - | -0.036 (0.015) | -2.325  (0.024 *) |
| Species*Sex  (white-fronted female) | -0.121 (0.537) | -0.225  (0.823) | -0.166 (0.528) | -0.315  (0.754) |

Two GLMs were used to analyse home range size using Gaussian error structure and identity link function. The first including only Sex, Species, and their interaction; and the second including a number of additional control variables (above). The two models were tested and including control variables was not found to improve the fit of the model (Analysis of Deviance, F = 2.371, df_large_ = 52, df_small_ = 49, p = 0.082). Duration refers to the number of days that a plover was tracked in the field; captivity, whether or not the bird was held until its previous mate re-mated; and number of relocations, the number of GPS recordings taken. Reference factor level, estimate standard errors and t values’ corresponding p values are in parentheses.

**Table S2.** Mean step length of male and female Malagasy plovers.

|  | Model 1 | | Model 2 | |
| --- | --- | --- | --- | --- |
|  | Estimate | t value | Estimate | t value |
| Intercept | 4.978  (0.171) | 29.202  (<0.001***) | 4.722  (0.339) | 13.912  (<0.001 ***) |
| Species  (white-fronted) | -0.598  (0.259) | -2.310  (0.025 *) | -0.471  (0.300) | -1.566  (0.123) |
| Sex  (female) | 0.315  (0.241) | 1.307  (0.197) | 0.330  (0.248) | 1.332  (0.189) |
| Captivity  (yes) | - | - | 0.091  (0.192) | 0.474  (0.638) |
| Duration | - | - | 0.008  (0.009) | 0.832  (0.409) |
| Species*Sex  (white-fronted female) | -0.647 (0.366) | -1.767  (0.083 .) | -0.677  (0.375) | -1.805  (0.077 .) |

Two GLMs were used to analyse home range size using Gaussian error structure and identity link function. The first including only Sex, Species, and their interaction; and the second including a number of additional control variables (above). The two models were tested and including control variables was not found to improve the fit of the model (Analysis of Deviance, F = 0.387, df_large_ = 56, df_small_ = 54, p = 0.681). Duration refers to the number of days that a plover was tracked in the field; and captivity, whether or not the bird was held until its previous mate re-mated. Reference factor level, estimate standard errors and t values’ corresponding p values are in parentheses.

**Table S3.** Breeding dispersal of male and female Malagasy plovers.

|  | Model 1 | | Model 2 | |
| --- | --- | --- | --- | --- |
|  | Estimate | t value | Estimate | t value |
| Intercept | 4.354 (0.336) | 12.960  (<0.001 ***) | 4.174 (0.791) | 5.276  (<0.001 ***) |
| Species  (white-fronted) | 0.160 (0.522) | 0.305  (0.761) | -0.086 (0.627) | -0.137  (0.892) |
| Sex  (female) | -0.051  (0.491) | -0.103  (0.918) | 0.006  (0.505) | 0.012  (0.991) |
| Number of relocations | - | - | 0.0434  (0.037) | 1.198  (0.237) |
| Captivity  (yes) | - | - | 0.044 (0.402) | 0.108  (0.914) |
| Duration | - | - | -0.020 (0.023) | -0.895  (0.375) |
| Species*Sex  (white-fronted female) | -0.242  (0.749) | -0.323  (0.748) | -0.161 (0.775) | -0.208  (0.836) |

Two GLMs were used to analyse home range size using Gaussian error structure and identity link function. The first including only Sex, Species, and their interaction; and the second including a number of additional control variables (above). The two models were tested and including control variables was not found to improve the fit of the model (Analysis of Deviance, F = 0.525, df_large_ = 52, df_small_ = 49, p = 0.667). Duration refers to the number of days that a plover was tracked in the field; captivity, whether or not the bird was held until its previous mate re-mated; and number of relocations, the number of GPS recordings taken. Reference factor level, estimate standard errors and t values’ corresponding p values are in parentheses.
